# Autophagy in Plasma Cell Pathophysiology

**DOI:** 10.3389/fimmu.2014.00103

**Published:** 2014-03-12

**Authors:** Laura Oliva, Simone Cenci

**Affiliations:** ^1^Division of Genetics and Cell Biology, San Raffaele Scientific Institute, Milan, Italy; ^2^Università Vita-Salute San Raffaele, Milan, Italy; ^3^Bone Pathophysiology Program (BoNetwork), Division of Genetics and Cell Biology, San Raffaele Scientific Institute, Milan, Italy

**Keywords:** antibody, autophagy, endoplasmic reticulum, multiple myeloma, plasma cell, proteostasis, unfolded protein response, XBP-1

## Abstract

Plasma cells (PCs) are the effectors responsible for antibody (Ab)-mediated immunity. They differentiate from B lymphocytes through a complete remodeling of their original structure and function. Stress is a constitutive element of PC differentiation. Macroautophagy, conventionally referred to as *autophagy*, is a conserved lysosomal recycling strategy that integrates cellular metabolism and enables adaptation to stress. In metazoa, autophagy plays diverse roles in cell differentiation. Recently, a number of autophagic functions have been recognized in innate and adaptive immunity, including clearance of intracellular pathogens, inflammasome regulation, lymphocyte ontogenesis, and antigen presentation. We identified a previously unrecognized role played by autophagy in PC differentiation and activity. Following B cell activation, autophagy moderates the expression of the transcriptional repressor Blimp-1 and immunoglobulins through a selective negative control exerted on the size of the endoplasmic reticulum and its stress signaling response, including the essential PC transcription factor, XBP-1. This containment of PC differentiation and function, i.e., Ab production, is essential to optimize energy metabolism and viability. As a result, autophagy sustains Ab responses *in vivo*. Moreover, autophagy is an essential intrinsic determinant of long-lived PCs in their as yet poorly understood bone marrow niche. In this essay, we discuss these findings in the context of the established biological functions of autophagy, and their manifold implications for adaptive immunity and PC diseases, *in primis* multiple myeloma.

## Introduction

The biology of plasma cell (PC) differentiation is a unique model for scientists to investigate the complex connections between metabolism, stress, proteome plasticity, and cellular renovation. In particular, the regulation of antibody (Ab) production is a valuable paradigm for the molecular wirings controlling protein folding and assembly in professional secretory cells. Moreover, the bone marrow PC niche, which provides lifelong Ab memory, depends on as yet incompletely understood intrinsic and environmental components, whose derangement is instrumental to the development of multiple myeloma.

We recently discovered an unanticipated essential function played by autophagy during PC differentiation, disclosing new links with endoplasmic reticulum (ER) homeostasis and Ab production. Moreover, autophagy emerges as an intrinsic requirement of long-lived PCs and long-term immunity (1). Scope of the present review is to discuss the newly identified role of autophagy in PC pathophysiology, in perspective of recently established autophagic functions across stress biology, cell differentiation, immunity, and cancer.

## The Stress of Plasma Cell Differentiation

Plasma cells are the terminal effectors of adaptive immunity endowed with the unique ability to secrete Abs capable of neutralizing pathogens and toxins. Upon encounter with antigens (Ags), B cells differentiate into short-lived PCs in secondary lymphoid organs (e.g., spleen and lymph nodes). Most of these effector cells die within few days. In addition, T cell-dependent responses induce the germinal center reaction, which generates a second wave of plasmablasts, secreting high-affinity, class-switched Abs, and capable of acquiring lifelong survival in dedicated bone marrow niches. Long-lived PCs maintain immunological memory of Ab-inducing Ags, yielding prompt protection against pathogens and their toxic products (2).

From a biological standpoint, PCs are professional secretory cells dedicated for massive synthesis, assembly, and secretion of Abs. To accomplish this mission, upon activation, B cells must reshape their proteome. To this aim, a powerful genetic program silences B cell identity, through the repression of genes encoding the transcription factors Pax5 and Bcl-6, and establishes PC function, inducing the transcriptional regulators IRF4 and PRDM1/Blimp-1 (3). Early during differentiation, XBP-1, a key ER stress transducer and transcription factor of the unfolded protein response (UPR), drives ER expansion to augment the folding capacity of this organelle and accommodate intensive immunoglobulin (Ig) synthesis in the secretory pathway (4, 5).

In addition to ER stress, other stresses are constitutive of full gear Ab production in PCs. For example, oxidative protein folding causes redox stress, counterbalanced by antioxidant responses (6, 7). After the blastic phase, in post-mitotic PCs, additional stress may ensue from the impossibility of diluting damaged organelles through cell division, as demonstrated in other non-dividing terminally differentiated cells (8). We found that PCs also experience profound *proteasome stress* (9). Indeed, although Ig-synthetic activity requires intense proteasome-dependent degradation of Ab byproducts, during their differentiation short-lived PCs display a progressive, remarkable reduction of proteasome expression, which leads to accumulation of poly-ubiquitinated proteins, at the expense of free ubiquitin – an additional stress referred to as *ubiquitin stress* (10) – and stabilization of proapoptotic factors (11, 12). This apparently paradoxical lack of adaptation may serve as a built-in mechanism to reduce the apoptotic threshold and limit PC lifespan and the duration of Ab responses (13). We also noted that PC differentiation confers exquisite sensitivity to proteasome inhibition, rendering PCs as sensitive to proteasome inhibitors as multiple myeloma cells, disclosing a general characteristic of PCs, rather than a feature of malignancy (9). Attenuating general protein synthesis by the otherwise toxic agent cycloheximide reduces proteasome sensitivity in differentiating plasmablasts, indicating protein synthesis as a key determinant of the proteolytic burden on proteasomes in PCs (14). Such a challenged protein homeostasis (*proteostasis*) may explain why the first-in-class proteasome inhibitor bortezomib reduced Ab responses (12) and attenuated autoAb-mediated pathology in a mouse model of lupus (15). Clearly, basic stress biology in PCs is instructive on putative targets against PC dyscrasias (see below).

## Autophagy: From Bulk Degradation to Selective Recycling

Autophagy is a highly conserved self-digestive strategy that envelops cytoplasmic contents in a double-membrane vesicle, the *autophagosome*, delivered to the lysosome (in animal cells) or to the vacuole (in plant and yeast cells) for subsequent degradation and recycling. The prime function of autophagy in unicellular organisms is to sustain cellular metabolism in conditions of nutritional starvation (16, 17). This metabolic role is conserved in metazoa, where autophagy is an essential source of energetic equivalents (18). An exemplar case, autophagy-incompetent newborn mice fail to resist the physiologic early neonatal starvation (19). Autophagy also provides building blocks for cellular renovation, and is crucially involved in differentiation and development (18, 20). In mammals, autophagy is essential for embryogenesis (21) and lineage differentiation, as demonstrated, for example, in adipocytes, erythrocytes, and lymphocytes (20).

By contrast with the ubiquitin–proteasome system, autophagy has long been viewed as a bulk non-selective process, with the only exception of chaperone-mediated autophagy. Its recently recognized capacity to ensure cellular quality control by clearing toxic and damaged macromolecules and organelles disclosed an unanticipated level of selectivity (22, 23). Selective autophagic degradation has been reported for a number of endogenous supramolecular structures: peroxisomes (*pexophagy*) (24), protein aggregates (*aggrephagy*) (25–27), ribosomes (*ribophagy*) (28), mitochondria (*mitophagy*) (29–31), lipid droplets (*lipophagy*) (32), secretory granules (*zymophagy*) (33), and midbody remnants after cytodieresis (34).

To target selected cargoes, autophagy makes use of adapter proteins acting as receptors. To mediate selective autophagy, these proteins must: (i) recognize substrates via a ubiquitin-binding activity; (ii) cross-link the cargo with the autophagic machinery via an LC3-interacting region; and (iii) polymerize (23, 35). Ubiquitination is thus used not only to convey individual proteins to the proteasome, but also for selective recognition by autophagic receptors, e.g., p62 and NBR1 during mitophagy and aggrephagy. While the prime tag for proteasomal degradation is a chain of ubiquitins covalently linked through their K48 lysine residues, K63-linked poly-ubiquitin tags may be preferentially associated with autophagic degradation, although additional post-translational modifications may contribute to direct cargoes to autophagy (23). In most cases, the ubiquitin ligases involved in autophagy remain to be identified. Hitherto established mammalian autophagic receptors include SQSTM1/p62, NBR1, optineurin, NDP52, and Nix. Adaptor proteins are also being characterized, which interact with autophagy receptors to recruit and assemble more Atg proteins, so as to shape the growing autophagosome (*phagophore*) around the cargo (36).

Virtually, all cellular membranes have been proposed to contribute to autophagosome biogenesis. Among them, the ER is an established membrane source for the phagophore (37). The ER may also undergo autophagic degradation: autophagic trimming of excess ER (*reticulophagy, ER-phagy*) counterbalances pharmacological stress-induced ER expansion in yeast (38). More recently, a mechanism mediating both mitophagy and ER-phagy has been described in HeLa cells (39). However, defining the physiological significance of reticulophagy in mammals is biologically relevant. As described below, our recent work on autophagy in PCs furthers this view by defining ER-phagy as an essential determinant of PC biology and Ab immunity (1).

## Roles of Autophagy in Innate and Adaptive Immunity

In the immune system, autophagy serves diverse innate and adaptive functions, including microbe clearance, Ag presentation, and the regulation of inflammation and lymphocyte development (40–42). The co-optation of autophagy to destroy intracellular microbes, i.e., *xenophagy*, already present in unicellular organisms (43), likely represents the most ancient form of immune defense. Xenophagy has been shown to restrict the growth of bacteria (*L. monocytogenes, S. flexnerii, S. typhimurium*) (42). The infectious phagosome is intracellularly recognized through internal toll-like receptor (TLR) signaling (44). Then, infected cells can promote phagosome–lysosome fusion or target cytosol-invading bacteria for autophagic degradation (36). A number of autophagic receptors, including SQSTM1/p62, NDP52, and optineurin, have been shown to specifically recognize ubiquitinated bacteria within the cytosol (23, 36), hence the idea that SQSTM1-like receptors (SLRs) constitute a new family of innate pattern recognizing receptors (45). Autophagy may play additional antimicrobial activities through SLRs, e.g., by generating microbicidal peptides via incomplete digestion of ribosomal protein precursors during *M. tuberculosis* infection (46). Autophagy also mediates viral recognition and destruction. For example, capsid proteins of the neurotropic Sindbis virus are degraded via p62-dependent autophagy (47).

Autophagy is also involved in the modulation of the inflammatory response. In particular, autophagy may both stimulate and inhibit the activity and output of the inflammasome. While basal autophagy prevents inflammation, e.g., by limiting mitochondrial generation of reactive oxygen species and the resulting inflammasome activation (48, 49), induction of autophagy can promote inflammation, mediating the inflammasome-dependent unconventional release of the *endogenous pyrogen*, IL-1β, which in turn can intensify autophagy (50). Autophagy may also yield negative feedback loops to prevent destructive inflammation, e.g., moderating IL-1β release by targeting inflammasomes and pro-IL-1β for degradation (51, 52).

Autophagy also serves adaptive immune functions, including the regulation of lymphocyte ontogenesis and homeostasis. Atg proteins have been shown to maintain normal numbers of CD4^+^ and CD8^+^ T cells, and fetal hematopoietic stem cells (41, 53, 54). First, Atg5^−/−^ bone marrow chimeric mice revealed defects in T cell development and peripheral homeostasis, and impaired activation-induced proliferation (55). Although activated T cells require autophagy, negative controls may come into play to temper autophagy, preventing detrimental effects. Indeed, components of the extrinsic apoptotic cascade, namely FADD and caspase 8, were found to limit autophagy by interacting with the Atg5–Atg12 complex, thereby sustaining viability of activated T cells (56). In following studies, the development of mature T cells was found to require an active negative control on the intracellular production of reactive oxygen species, which in turn relies on efficient mitophagy (55, 57). Such mitochondrial quality control maintains mature naïve T cell homeostasis through Beclin-1 stabilization by the class III phosphoinositide-3 kinase Vps34 (58, 59). Moreover, in activated T cells, autophagy is induced to maintain ATP levels, proliferation, and the release of cytokines (60).

Autophagy is also important for B cell development: irradiated Rag1^−/−^ recipients repopulated with fetal liver progenitors lacking the essential autophagic factor Atg5 have low counts of peritoneal B-1 B cells, due to defective transition of pro- to pre-B cells (61). Moreover, mice with conditional deletion of *Atg5* in mature B cells (*Atg5^f/f^*CD19-Cre) show normal numbers of mature B lymphocytes and a normal ratio of marginal zone to follicular B cells, but reduced maintenance of B-1a cells in the periphery (1, 61).

A number of studies have implicated autophagy in different Ag presentation pathways. The delivery of exogenous Ags for MHC class II presentation to CD4^+^ T cells has been shown to depend on autophagy (62). Indeed, MHC class II-loading compartments receive continuous input from autophagosomes, and autophagy has been shown to positively control CD4^+^ T cell priming (63–65). Moreover, thymic epithelial cells deliver self Ags to MHC class II-loading compartments through the autophagic machinery. This task is essential to build self-tolerance, as its disruption leads to defective elimination of autoreactive T cells and autoimmunity (66). Furthermore, autophagy has been shown to mediate CD8^+^ T cell priming *in vivo* through cross-presentation of phagocytosed Ags, normally routed through the MHC class II pathway, on MHC class I (67, 68). However, autophagy is not a universal Ag-presenting pathway, as we proved it dispensable for presentation by B cells to cognate T cells in the germinal center (see below) (1).

We hypothesized that autophagy may play an additional adaptive immune function in terminal PC differentiation, based on the specific biology of Ab-secreting cells (9). First, PC differentiation is expected to require a high degree of proteome plasticity. In support of this notion, we had generated quantitative evidence that both protein translation and degradation increase remarkably in primary activated B cells (14). Second, we had observed that such an increased demand for protein degradation is not met by a corresponding increase in proteasome capacity, which instead decreases dramatically (11, 12, 14), and reasoned that this would call for complementary protein degradation routes. Having the capacity to compensate for proteasome insufficiency (69), autophagy was an obvious candidate. Third, most, if not all, stresses experienced by PCs are known to be relieved by autophagy (9, 22). The following paragraphs illustrate our findings, unveiling the crucial role served by autophagy in the differentiation, function, and viability of PCs, required for humoral immunity, and the underlying mechanism, linking ER homeostasis with Ig synthesis and energy metabolism.

## Autophagy Sustains Ab Responses and is Essential to Long-Lived PCs

When we assessed overall autophagic activity in differentiating PCs, we found strong induction of autophagy following B cell activation, both *ex vivo* and *in vivo*. In keeping with a developmental program, similar to UPR transcripts, Atg mRNAs increased concertedly during PC differentiation. The use of GFP-LC3 transgenic mice revealed intense autophagy also in long-lived bone marrow PCs (1). Encouraged by these observations, to assess the functional relevance of autophagy in PC ontogenesis, we first investigated Ab responses in *Atg5^f/f^*CD19-Cre mice. These mice showed reduced IgM and IgG responses in both T-independent and T-dependent immunization experiments, demonstrating a positive role of autophagy in Ab responses mediated by short-lived PCs. A parallel independent study confirmed these findings, by showing significantly diminished Ab titers in the same mouse model during Ag-specific immunization, parasitic infection, and mucosal inflammation (70).

Inspired by the observation of high autophagic activity also in long-lived PCs, we then asked whether Atg5 is required for long-term humoral immunity, by assessing if *Atg5^f/f^*CD19-Cre mice show defects in bone marrow PC populations. These mice had normal bone marrow PC counts, apparently arguing against a role for autophagy in long-lived PCs. However, the genomic quantification of Cre-mediated deletion of *Atg5* disclosed that while in splenic B cells most *Atg5* alleles had undergone Cre-dependent recombination, bone marrow PCs displayed normal amounts of the non-deleted allele. Hence, an efficient Darwinian selection for autophagy-competent PCs had occurred, demonstrating that autophagy is absolutely required to establish or maintain long-lived PCs. Moreover, despite a normal size of the bone marrow PC pool, *Atg5^f/f^*CD19-Cre mice revealed a defect in long-term Ab immunity, as they had virtually absent Ag-specific long-lived PCs in the bone marrow 11 months after T-dependent immunization. Altogether, the data establish autophagy as a novel determinant of the PC memory compartment (1).

Being essential to generate class-switched and high-affinity memory B cells and long-lived PCs, we also checked the germinal center reaction in NP-CGG-immunized *Atg5^f/f^*CD19-Cre mice, but found it normal (1). This evidence mapped the requirement of autophagy specifically to PCs. Moreover, since the germinal center response requires Ag presentation by B cells to cognate T cells, this data demonstrated that autophagy is dispensable for soluble Ag presentation in B cells, in spite of its Ag-presenting role in other contexts (discussed above).

The above findings further our understanding of the intrinsic molecular competence required for PCs to achieve extended survival in the bone marrow (2). Hitherto recognized components of such competence comprise the chromatin modifier Aiolos (71), the transcriptional regulators Blimp-1 (72) and XBP-1 (73), and the anti-apoptotic molecule Mcl-1 (74). The identification of autophagy as a novel molecular requirement of bone marrow PCs and of long-lived humoral immunity is in keeping with its established capacity to grant extended survival to quiescent progenitors and highly specialized terminally differentiated cell types, such as neurons (75).

An important matter of future investigation is the precise level at which autophagy is required in memory PC ontogenesis, i.e., the survival of non-resident long-lived plasmablasts, their migration to the bone marrow, or the maintenance of resident long-lived PCs in the medullary niche. Moreover, it would be interesting to determine if autophagy also plays a role in maintaining the other memory compartment of B cell immunity, i.e., non-Ig-secreting memory B cells.

## Autophagy Contains PC Differentiation and Ab Production through Selective ER-Phagy

The easiest conceivable explanation for autophagy sustaining Ab immunity was to hypothesize it to be required for PC differentiation. Confuting this hypothesis, Atg5-deficient B cells apparently underwent normal PC differentiation (1). The exact molecular role of autophagy in developing PCs was gaged by an unbiased comparison of the proteome of autophagy-competent vs. incompetent activated B cells by stable isotope labeling in cell culture (SILAC). Importantly, the proteome of differentiating PCs was completely labeled in as little as 3 days upon activation, not only enabling this approach, but also convincingly demonstrating the highest proteome plasticity inherent to this differentiation program. SILAC proteomics of Atg5^−/−^ PCs revealed a selective and rather exclusive expansion of the ER proteome, including Igs. An autophagic regulation of the size of the ER was demonstrated by two independent electron microscopy (EM) approaches: classical EM and an EM cytochemistry technique designed to stain and unbiasedly quantify the ER (76). Short treatment with distal autophagy inhibitors was sufficient to increase ER proteins in wild type differentiating PCs, unveiling the first case of physiologic reticulophagy in mammals (1).

**Figure 1:**
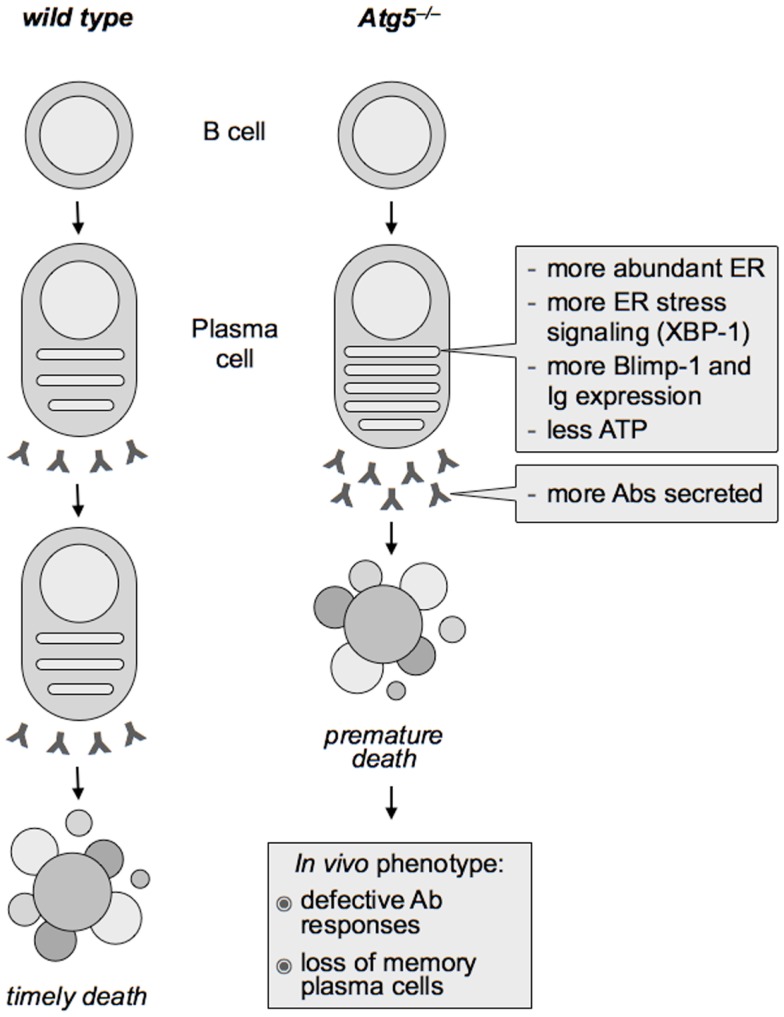
**Effect of Atg5 deficiency on plasma cell differentiation and Ab immunity**. In differentiating plasma cells, autophagy limits the expansion of the endoplasmic reticulum (ER), providing the first physiological case of mammalian ER-phagy. As a result, Atg5^−/−^ plasma cells show more abundant ER than wild type counterparts and more intense ER stress signaling (including higher expression of the essential plasma cell transcription factor, XBP-1), which in turn enhances the expression of the key plasma cell transcriptional regulator PRDM1/Blimp-1 and of immunoglobulins (Ig). Hence, despite increased Ig production, autophagy-deficient plasma cells have less ATP and live a shorter life, yielding defective Ab responses *in vivo*.

Attesting to the functional relevance of the identified autophagic regulation of the ER in PC differentiation, Atg5^−/−^ PCs had higher UPR signaling than wild type PCs, associated with higher expression of Blimp-1 and Ig transcripts, indicating that autophagy restricts the expression of two key determinants of PC differentiation, XBP-1 and Blimp-1, and of Igs. Providing mechanistic insight, pharmacological ER stress in wild type differentiating PCs was sufficient to increase Blimp-1 and Ig expression beyond the putatively maximal levels associated to PC differentiation. As a result, Atg5^−/−^ PCs translated, assembled, and secreted more Abs over time, disclosing an unsuspected regulatory circuit of PC function negatively controlled by autophagy (1, 75) (see Figure 1). It will be important to dissect the mechanisms underlying ER-phagy during PC differentiation.

In cellular models of protein folding diseases, autophagy has been shown to dispose of polymeric misfolded protein aggregates in the ER, constituting an alternative form of ER associated degradation (ERAD) (77, 78). Noticeably, instead, in differentiating PCs, autophagy does not serve a similar quality control function in the secretory pathway, nor does it remove dysfunctional ER, as Atg5^−/−^ PCs show normal Ig assembly, and do not accumulate Ig aggregates, but rather display higher capacity in their expanded secretory apparatus (1).

These findings imply that PCs are programed to become more productive Ab factories than actually observed, with autophagy acting as a physiologic brake on their differentiation and function. How is this reconciled with the defective Ab responses mounted by *Atg5^f/f^*CD19-Cre mice? This paradox is solved by the observation that Atg5^−/−^ PCs have less ATP and live a shorter life than wild type counterparts. Hence, autophagy accomplishes a sensible trade-off between viability and function, setting Ab production to sustainable levels (1, 75).

Can the higher Ig-secreting potential of PCs be demonstrated *in vivo*? A proof-of-principle experiment was the immunization with the T-independent hapten NP-ficoll, which, in our hands, yielded higher anti-NP Ig titers in *Atg5^f/f^*CD19-Cre mice, despite normal PC counts, in line with the higher secretory activity of Atg5^−/−^ PCs observed *ex vivo*. Unlike other Ags, NP-ficoll has been shown to persist and cause continual B cell activation (79). Hence, repeated rounds of PC differentiation may have surpassed the otherwise dominant impact of reduced PC viability on Ab titers, witnessing the hypersecretory effect of Atg5 deficiency at the single PC level (1, 75).

The discovery of a novel autophagy-centered regulatory network balancing PC activity and survival *in vivo* implies an unsuspected plasticity of Ab responses, potentially exploitable to tune their duration and intensity. A number of immune signaling molecules can regulate autophagy (41, 45), supporting this possibility, and offering opportunities to search for molecular targets to modulate Ab responses, of therapeutic use against autoimmune diseases.

## Autophagy in Multiple Myeloma

A matter of intense scientific debate, the role of autophagy in cancer is complex. Genetic defects of autophagy have been linked with tumorigenesis, establishing the notion that autophagy is a tumor suppressive pathway (80). Oncosuppressive mechanisms of autophagy include protection against the accumulation of oncogenic mutations (81–83) and reactive oxygen species, mainly through mitochondrial homeostasis, and reduction of necrosis and local inflammation (84). While in healthy cells, autophagy may suppress tumor initiation, established cancers may subvert autophagy to cope with intrinsic (e.g., metabolic), environmental (e.g., hypoxic), or pharmacological stress (e.g., induced by cytotoxic agents). This may explain why pharmacological inhibition of autophagy may be beneficial against cancer, being toxic to tumor cells and sensitizing them to chemotherapy (80, 85).

Multiple myeloma is a valuable model to investigate the role of autophagy in cancer, especially in perspective of integrated cancer proteostasis. Indeed, myeloma represents the paradigmatic neoplasm responsive to proteasome inhibitors, prototypical negative proteostasis regulators, although a substantial proportion of patients fail to respond, and resistance inevitably ensues (9, 86–88). We demonstrated that the exquisite proteasome sensitivity of normal and malignant PCs stems from an unfavorable ratio between proteasome workload and overall capacity (9, 11, 12), and termed this feature *proteostenosis* (13). Moreover, we found that myelomas with the highest sensitivity to proteasome inhibition are those expressing fewer active proteasomes in spite of the highest degradative workload, both features being causal to such inherent vulnerability (89). Noticeably, the degradative burden is a relatively neglected source of cellular stress, particularly in cancer (90). In myeloma, we found that recent protein synthesis saturates the limited capacity of the ubiquitin–proteasome system, causing the buildup of ubiquitin conjugates, and is a crucial determinant of proteasome stress (14). It is noteworthy that *lymphoplasmocytic lymphoma* (*Waldenstrom’s macroglobulinemia*), another B cell cancer known to produce high levels of IgM, also proved vulnerable to proteasome inhibition (91–93), and the clinical use of bortezomib yielded encouraging results (94, 95).

Autophagy and the ubiquitin–proteasome system are integrated strategies that cooperate to maintain cellular proteostasis (69). This notion is sufficient to predict that multiple myeloma and *Waldenstrom’s macroglobulinemia* may be as dependent on autophagy as they are on the ubiquitin–proteasome system. This prompted different laboratories to target autophagy in order to overcome resistance to proteasome inhibitors and achieve myeloma cell death, with controversial results. While the blockade of autophagy appears toxic against human myeloma lines, the combined inhibition of the proteasome and autophagy may be synergic or antagonistic, depending on the molecular level of autophagic inhibition (88, 96–98). Experimental discrepancies may be partly explained by the recent discovery of a vital circuit blocking autophagy, disclosing that deregulated autophagy can turn maladaptive in multiple myeloma cells. In brief, human myeloma lines depend for their survival on IRF4, which, through a caspase 10-dependent mechanism, prevents excessive autophagy from executing non-apoptotic myeloma cell death (99). This data suggests that autophagy may play both adaptive and maladaptive roles in myeloma, depending on its intensity, or its targets. A better understanding of the basal, adaptive function of autophagy in myeloma cells is needed to harness this pathway against cancer. Our identification of a new homeostatic function of autophagy, essential for the maintenance of long-lived PCs in the bone marrow, the normal counterpart of multiple myeloma (1, 75), prompts to test if PC tumors are at least as dependent on autophagy for their survival, and provides a framework for dissecting the precise function of autophagy in normal and malignant bone marrow PCs. The diverse homeostatic activities of autophagy in different lineages and diseases hitherto defined suggests that PC-specific, and possibly myeloma-specific, autophagic circuits may be identified, linking organelle homeostasis, stress responses, and energy metabolism, to disclose new molecular therapeutic targets.

## Conflict of Interest Statement

The authors declare that the research was conducted in the absence of any commercial or financial relationships that could be construed as a potential conflict of interest.

## Abbreviations

Ab, antibody; Ag, antigen; EM, electron microscopy; ER, endoplasmic reticulum; ERAD, ER associated degradation; Ig, immunoglobulin; PC, plasma cell; SILAC, stable isotope labeling in cell culture; SLR, SQSTM1-like receptor; TLR, Toll-like receptor; UPR, unfolded protein response
